# Editorial: The role of metabolic syndrome and disorders in cardiovascular disease, volume II

**DOI:** 10.3389/fendo.2025.1639896

**Published:** 2025-06-18

**Authors:** Carmine Izzo, Paola Di Pietro, Jessica Gambardella, Albino Carrizzo

**Affiliations:** ^1^ Department of Medicine, Surgery and Dentistry “Scuola Medica Salernitana”, University of Salerno, Baronissi, Italy; ^2^ Cardiology Unit, University Hospital “San Giovanni di Dio e Ruggi d’Aragona,”, Salerno, Italy; ^3^ Department of Advanced Biomedical Sciences, “Federico II” University, Naples, Italy; ^4^ Department of Medicine, Einstein-Sinai Diabetes Research Center, The Fleischer Institute for Diabetes and Metabolism, Einstein Institute for Aging Research, Albert Einstein College of Medicine, New York, NY, United States; ^5^ Vascular Physiopathology Unit, IRCCS Neuromed, Pozzilli, Italy

**Keywords:** metabolic syndrome, risk factors, cardiovascular disease, biomarkers, cardiovascular complications

The global burden of cardiovascular disease (CVD) remains persistently high, with a marked increase in incidence attributable to metabolic disorders such as obesity, insulin resistance, and dyslipidemia. Metabolic syndrome (MetS), characterized by a cluster of risk factors including central obesity, hyperglycemia, dyslipidemia, and hypertension, plays a pivotal role in the pathogenesis and progression of CVD. This second volume of the Research Topic “*The Role of Metabolic Syndrome and Disorders in Cardiovascular Disease*” builds upon prior knowledge, further elucidating the complex interplay between metabolic dysfunction and cardiovascular outcomes. The contributions in this Research Topic explore diverse mechanistic, clinical, and epidemiological perspectives, offering new insights with translational and preventive relevance.

A prominent theme across the studies is the prognostic value of metabolic indices and biomarkers in cardiovascular risk stratification. In a retrospective cohort, Zhao et al. demonstrated that the triglyceride-glucose (TyG) index, a marker of insulin resistance, independently predicted major adverse cardiovascular and cerebrovascular events (MACCE) in patients with coronary heart disease (CHD) and coexisting depression. Similarly, the ankle-brachial index (ABI) was examined in two complementary studies. Wu et al. found an inverse association between ABI and erectile dysfunction (ED), suggesting vascular dysfunction as a key mediator, while Wang and Ni confirmed that ED may serve as a clinical indicator of peripheral arterial disease (PAD), reinforcing the shared vascular basis of these conditions.

Several articles evaluated novel risk markers and indices with cardiometabolic relevance. Non-HDL-cholesterol to HDL-cholesterol ratio was strongly associated with arterial stiffness and may outperform traditional lipid measures in predicting vascular damage (Guo et al.). In another population-based study, Zhou et al. identified a positive association between blood ethylene oxide levels and MetS, underlining the possible contribution of environmental toxins to metabolic dysfunction.

The impact of cardiometabolic risk on mortality was comprehensively analyzed in heart failure populations. Zhou et al. categorized patients with chronic heart failure into distinct metabolic obesity phenotypes and demonstrated that metabolically unhealthy individuals, regardless of BMI, faced elevated mortality risks. Interestingly, the so-called “obesity paradox” appeared to be modified by age and sex. This nuanced analysis underscores the importance of metabolic profiling beyond body weight alone.

Cardiorenal and cerebrovascular interactions also emerged as important considerations. Wang et al. conducted a meta-analysis of sodium-glucose cotransporter 2 inhibitors (SGLT2i), confirming their efficacy in reducing heart failure, stroke, and all-cause mortality—further validating their role in high-risk patients with type 2 diabetes. Complementing these findings, Wang and Meng showed that higher scores on the Life’s Essential 8 cardiovascular health metrics correlated with lower uric acid levels, suggesting a link between lifestyle-driven CV health and risk for hyperuricemia—a known contributor to both renal and vascular complications.

Sex-specific and hormonal influences on metabolic and vascular outcomes were another central focus. Testosterone deficiency impacts inflammatory markers in obese male mice, revealing increased IL-6 expression and potential implications for male-specific CVD risk (Malagon-Soriano et al.). In a related experimental study, physical exercise improved lipid metabolism and gut microbiota composition in ovariectomized rats, highlighting a protective role in postmenopausal women (Song et al.).

Psychosocial and cognitive dimensions of cardiometabolic disease were examined by Chen et al., who found that MetS was significantly associated with cognitive impairment among patients with bipolar disorder, emphasizing the need for holistic care approaches. Similarly, Mehran et al. explored the predictive value of TyG-related indices for MACCE in hypertensive patients with CHD, suggesting their utility in behavioral and pharmacologic risk stratification.

The importance of precision medicine was highlighted in studies exploring phenotype stratification. Huang et al. examined metabolically healthy and unhealthy obesity in adolescents, finding that BMI alone is insufficient to assess metabolic risk. Complementing this, Zeng et al. described the independent contributions of visceral adiposity to subclinical atherosclerosis in Chinese adults, regardless of overall obesity.

Clinical management perspectives were advanced in multiple articles. Zhou et al. found that serum uric acid was positively associated with pulse wave velocity, adding evidence to its role as a modifiable vascular risk marker. Wang et al. also explored the relationship between C-peptide levels and stroke in diabetic individuals, finding a nonlinear association that might inform future therapeutic thresholds.

From a population health standpoint, Wang and Meng used NHANES data to establish a robust inverse relationship between cardiovascular health (assessed by LE8) and hyperuricemia, supporting the use of preventive lifestyle metrics to mitigate metabolic burden. In a related NHANES-based analysis, Wu et al. and Wang et al. provided complementary evidence on the link between ABI, ED, and PAD, reinforcing the systemic nature of metabolic vascular damage.

Lastly, Wang et al. contributed an innovative study on the bidirectional relationship between serum 25(OH)D levels and CVD, applying Mendelian randomization and reinforcing the vitamin D hypothesis in cardiovascular prevention.

Taken together, the articles in this volume offer a comprehensive and multidimensional perspective on how metabolic syndrome and its components intersect with cardiovascular pathophysiology. They reinforce the critical need for early detection, personalized risk profiling, and integrated therapeutic approaches that bridge endocrinology and cardiology. We extend our gratitude to the contributing authors, peer reviewers, and editorial team for enriching this Research Topic with robust and impactful science. We hope this Research Topic stimulates further interdisciplinary collaboration and informs future research and clinical innovation in cardiometabolic health.

